# Supplementary material for: Determinism and Contingency Shape Metabolic Complementation in an Endosymbiotic Consortium
Source: Front Microbiol. 2017 Nov 22;8:2290. doi: 10.3389/fmicb.2017.02290 (PMC5702781; doi:10.3389/fmicb.2017.02290)
Supplement: Supplementary Text S1 — Extended results. The text includes four sections, two including details about the reconstruction and metabolic capabilities of the endosymbiont individual models. The other two sections correspond to the estimations of the proteome composition of C. cedri and to the construction of the meta-biomass. [file TextS1.PDF]

Supplementary Text S1 - Extended Results

Genome-scale metabolic reconstruction of the cedar aphid (*Cinara cedri*) endosymbionts: *Buchnera aphidicola* BCc and *Serratia symbiotica* SCc

In order to reconstruct the metabolic model of *S. symbiotica* SCc, we firstly identified orthologous genes in the *Escherichia coli* K12 MG1655 genome. Of the 712 annotated genes in *S. symbiotica* SCc, 665 were found to have orthologs in *E. coli*. We then filtered these genes to keep only those with enzymatic activities, thus yielding a total of 236 genes. Additionally, 47 genes for which no ortholog was found were analysed manually, thus identifying 2 genes encoding enzymatic activities: the SCc_569 gene, which encodes an NH_3-dependent NAD^+ synthase (EC 6.3.5.1 and EC 6.3.1.5), and the SCc_469 gene, which codes for the riboflavin synthase beta subunit (EC 2.5.1.9). From the list of 236 metabolic genes, and using the *E. coli i*AF1260 metabolic model as a reference, we constructed the GPR association table. This table allowed the inference of the reactions present in the metabolism of *S. symbiotica* SCc, which were extracted from the BiGG database (Schellenberger et al., 2010)□. This first version of the network included 208 reactions, 17 gene-associated transporters and 5 diffusion transport reactions.

***Table 1. Description of the primary structure of the* S. symbiotica *SCc i*SCc236 *and* B. aphidicola *BCc i*BCc98 *metabolic models*.**

**Element**	***i*SCc236**	***i*BCc98**
Metabolic genes	236	98
Reactions associated with a gene †	209	95
Orphan reactions	11	8
Transporters associated with a gene †	30	5
Orphan transporters	49	58
Metabolites (intracelular/extracelular)	267 / 78	155 / 65
Exchange fluxes	78	65

† *The count of reactions and transporters associated with a gene also includes spontaneous reactions and excludes the biomass flux.*

The consistency analysis performed using the UM approach allows the detection of 8 gaps present in diverse metabolic pathways. These gaps were completed manually by adding the following orphan reactions: i) two activities in the coenzyme A synthesis pathway (EC 4.1.1.36 and EC 6.3.2.5); ii) two activities in the tetrahydrofolate (THF) biosynthesis pathway (EC 4.1.2.25 and EC 3.6.1.-); iii) one activity in the pyridoxal phosphate synthesis pathway (EC 1.4.3.5); and iv) three activities involved in the synthesis of various membrane components. Combining the analysis of UMs with the prediction of minimal media, a set of 19 compounds was identified, which are required to be exchanged with the host: 11 of these compounds must be imported and include amino acids and precursors thereof, and 8 are secondary products that must be excreted. Consequently, 19 orphan transporters, and their corresponding exchange fluxes, were included. The transport reactions included some lacking a codifying gene and are defined as orphan reactions. Finally, since it has been suggested that a symbiotic a role of *S. symbiotica* SCc is the provision of vitamins and cofactors (Lamelas et al., 2011a, 2011b)□, 24 transport reactions were added to allow the export of these biosynthetic end-

products. The resulting GEM contained 236 genes encoding 239 enzymatic reactions and 35 transporters, and was named *i*SCc236 (see table 1). Moreover, *i*SCc236 includes 8 and 19 orphan reactions and transporters, respectively, that are essential for the production of biomass components (see supplementary table S1 for a complete description of the model).

Regarding *B. aphidicola* BCc, the revision of the published GEM (Belda et al. 2012)□ resulted in the removal of 8 genes, for one of the following three reasons: i) the enzymatic activity was found to be out of the scope of the model, *i.e.* the reaction is part of a process not included in the model; ii) the enzymatic activity was isolated and blocked, and there was no evidence supporting the presence of that reaction; iii) the gene was wrongly annotated and the enzymatic activity does not contribute to the organism's metabolism. Complementarily, we identified two genes that were not included in the previous version, encoding activities involved in the synthesis of lipoate (see supplementary table S2). Since the previous version did not include an extracellular compartment, nor transport reactions, 62 metabolites and 62 transport reactions were included in the model. Since no gene-associated transporters are known for *B. aphidicola* BCc (Charles et al., 2011)□, all transport reactions are considered orphan reactions, except for the 5 cases where transport is known to occur via diffusion. Finally, we revised the biomass equation and added 9 amino acids, the 4 deoxyribonucleotides, and 16 cofactors and coenzymes that were not present in the original version of the model (see supplementary table S2).

The new version of the metabolic model of *B. aphidicola* BCc was then named *i*BCc98, and it includes 98 genes encoding 95 enzymatic activities, and 8 orphan reactions; 6 of the orphan reactions are represented by the following 3 enzymatic activities: two transaminase activities (EC 2.6.1.42 y EC 2.6.1.58) and one deaminase (EC 4.3.1.19). Regarding the two former, some experiments have suggested that these reactions are catalysed by the host (Hansen and Moran, 2011)□. On the other hand, several alternatives have been suggested to explain the activity EC 4.3.1.19 (Douglas et al., 2002)□. Similarly, two other orphan reactions were added, corresponding to the activities EC 2.1.1.197 (malonyl-CoA methyltransferase) and EC 2.3.1.47 (8-amino-7-oxononanoate –8AONN– synthase), both involved in the 8AONN synthesis pathway. On the other hand, the model predicts that *S. symbiotica* SCc can synthesize tryptophan from anthranilate, which has been shown to be provided by *B. aphidicola* BCc in a paradigmatic case of metabolic complementation (Gosalbes et al., 2008; Lamelas et al., 2011a; Manzano-Marín et al., 2016; Martínez-Cano et al., 2015)□. Another case of metabolic complementation between the two symbionts predicted by the model occurs in the biotin synthesis pathway, which takes place via the import of the precursor 8AONN, produced by *B. aphidicola* BCc, as recently suggested (Manzano-Marín et al., 2016)□. Finally, *i*SCc236 predicts that *S. symbiotica* SCc requires diverse inorganic compounds and five precursors for the synthesis of cofactors. Oxygen is present within the first group, suggesting the organism might be a strict aerobe or microaerophile. Here, oxygen dependency is not an energetic requirement, but a necessity for the oxidation of ubiquinol, generated during the *de novo* synthesis of pyrimidines. Moreover, oxygen is used as electron acceptor during the synthesis of siroheme, which is an essential cofactor for the assimilation of sulfur during cysteine synthesis. Finally, predictions indicate that the *S. symbiotica* SCc metabolic network may use both glucose and fructose as carbon sources with identical consequences.

On the other hand, *i*BCc98 predicts that *B. aphidicola* BCc must import all deoxynucleotides, nucleoside triphosphates and all cofactors, as a consequence of its extreme genome reduction. Despite

the well accepted symbiotic role of *B. aphidicola* BCc in the provision of amino acids to the host and co-primary endosymbiont, 8 amino acids need to be incorporated, besides the tryptophan co-synthesis case explained above. Furthermore, this organism lacks phosphotransferase systems (PTS) for the transport and phosphorylation of glucose. According to the model, the intake of carbon sources must take place through the import of phosphorylated sugars, such as glucose 6-phosphate, ribulose 5-phosphate and fructose 6-phosphate. Given the fact that the other compounds need to be converted to glucose 6-phosphate for their metabolic usage, *i*BCc98 only includes transport of this metabolite as a carbon source.

Table 2. Essential components of the minimal medium predicted by* i*SCc236 and* i*BCC98.

**Class**	***S. symbiotica* SCc *i*SCc236**	***B. aphidicola* BCc *i*BCc98**
**Nucleotides**	Hypoxanthine	dATP, dCTP, dGTP, dTTP, AMP, GTP, CTP, UTP
**Amino acids**	**Ala,** Arg, **Asn, Gln,** His, Ile, Leu, Lys, Met, Phe, Pro, Thr, **Ser, Tyr,** Val, anthranilate	**Ala, Asn,** Asp, Cys, **Glu, Pro, Ser, Tyr,** Trp, ornithine, homocysteine
**Coenzymes**	Porphobilinogen, thiamine, 8AONN, (R)-pantothenate, shikimate	$NADP^+$, NAD^+, FAD, riboflavine, ubiquinone, pyridoxal 5-phosphate, thiamine 2-phosphate, malonyl-CoA, THF, S-adenosylmethionine
**Carbon source**	D-glucose / D-fructose	D-glucose 6-phosphate
**Inorganic compounds**	Zn^{2+}, SO_4^{2-}, Fe^{2+}, K^+, O_2, HPO_4^{2-}	H_2S, Fe^{2+}, K^+, HPO_4^{2-}

** Amino acids in bold are provided by the host.*

In-silico predicted biosynthetic and energetic capabilities

For each model, the metabolic capabilities were predicted using constraint-based modeling (for implementation details see Materials & Methods in the main text and the extended version in supplementary Text S2). Figure 2 represents the relation between the compounds synthesized by *S. symbiotica* SCc and *B. aphidicola* BCc, and those compounds required as precursor for each biosynthesis. Several of these conversions occur in a single step, such as the deamination of glutamine and asparagine producing glutamate and aspartate, respectively, and the dehydromethylation of serine producing glycine. In this organism, the pathway for the biosynthesis of cysteine is complete, which is produced from serine and sulphate. Tryptophane is, as mentioned previously, a paradigmatic case of metabolic complementation between endosymbionts, where the genes for its synthesis are separated in the two separate organisms (Gosalbes et al., 2008). As a consequence, *B. aphidicola* BCc synthesizes anthranilate from chorismate, which is produced from erythrose 4-phosphate, and *S. symbiotica* SCc produces tryptophane from anthranilate. The case of lysine is particularly interesting, since the genome of *S. symbiotica* SCc encodes all activities of the pathway but the last one, which transforms *meso*-diaminopimelate in lysine, suggestive of metabolic complementation (Lamelas et al.

2011a). However, *meso*-diaminopimelate is also necessary for the synthesis of peptidoglycan, and the complete lysine synthesis pathway is conserved in *B. aphidicola* (see later), which could indicate that this complementation does not occur.

Despite the reduced capabilities for amino acid biosynthesis, *i*SCc236 predicts the capability to synthesize all nucleic acid components. Particularly, it predicts the synthesis of purines from glutamine and hypoxanthine. This is in disagreement with previous results suggesting an inability to synthesize IMP (Lamelas et al., 2011a)□, since the *S. symbiotica* SCc genome includes the gene SCc_272, encoding the hypoxanthine phosphoribosyltransferase activity EC 2.4.2.8, which allows the synthesis of IMP from hypoxanthine and PRPP (which is synthesized in the pentose phosphate pathway, conserved in this organism). Moreover, the genome and the metabolic model of *S. symbiotica* SCc indicates that it has retained the ability to synthesize several cofactors. Indeed, the ability to synthesize riboflavin (vitamin B2) has been suggested as a fundamental role for the establishment of *S. symbiotica* SCc as a co-obligate endosymbiont in the Lachninae family (Manzano-Marín and Latorre, 2014)□. However, genome reduction has effectively disrupted some biosynthetic pathways, as in the case of NAD^+, where only the two last activities are encoded in the *S. symbiotica* SCc genome, indicating the need for metabolic complementation with the host. Another case of pathway disruption is the one mentioned above, where the synthesis of biotin in *S. symbiotica* SCc takes place from the metabolite 8AONN, which has been proposed to be complemented by *Buchnera* (Manzano-Marín et al., 2016). Another important case is the one regarding THF biosynthesis, which requires chorismate as a precursor and, consequently, it has been suggested as a metabolic requirement of *S. symbiotica* SCc (Lamelas et al., 2011a)□. However, the two last steps in the synthesis of chorismate (EC 2.5.1.19 and 4.2.3.5) are conserved in this organism, suggesting that shikimate, not chorismate, must be imported for the synthesis of THF and derivatives. Moreover, since the genomes of seven free-living *Serratia* species encode the activities for chorismate biosynthesis from erythrose 4-phosphate (last seen in Metacyc on November 15th, 2016), the division of shikimate biosynthesis in *B. aphidicola* BCc and its use in *S. symbiotica* SCc to synthesize chorismate, this pathway might represent another event of metabolic complementation previously undescribed.

Bioenergetically, the genome of *S. symbiotica* SCc includes all genes for glycolysis, which produces two molecules of pyruvate, ATP and NADH per molecule of glucose or fructose. However, in contrast with its free-living and symbiotic relatives, it has lost the Krebs cycle genes. Therefore, the pyruvate produced by glycolysis is fermented to produce two additional ATP and acetate molecules, the latter then being excreted. The maximum yield of ATP by substrate-level phosphorylation is, thus, 4 ATP molecules per glucose molecule. In addition to these, 4 NADH molecules are produced per glucose molecule, which can be used to generate 2 additional ATP molecules by the respiratory chain (by pumping 10 protons, where 6 generate force for the production of ATP, and 4 compensate intracellular processes), whose genes are present in *S. symbiotica* SCc. Altogether, *i*SCc236 predicts 6 ATP mols per glucose mol, which is considerably lower to the one predicted by the *E. coli* model *i*AF1260, of 23.5 ATP mols per glucose mol, largely due to the activity of the Krebs cycle. The model of *B. aphidicola* BCc includes (non-trivial) biosynthetic pathways for eight amino acids, and several substantial differences with close relatives. For instance, *i*BCc98 predicts the production of arginine from ornithine, which needs to be imported, in contrast to the case for *B. aphidicola* BAp from *A. pisum*, which contains a complete pathway for arginine biosynthesis. Moreover, *B. aphidicola* BCc

has also lost the ability to synthesize cysteine and alanine, both non-essential amino acids. A common trend in the genome reduction process of this organism has been the loss of transaminases, which affect the last steps in the biosynthesis of phenylalanine and branched amino acids. This activity may be complemented by the host or be catalysed by a lowly specific enzyme . As mentioned above, *B. aphidicola* BCc requires complementation in the biosynthetic pathways for tryptophan and biotin, and lacks the ability to synthesize cofactors and nucleotides, which then must be provided by the host or the other member of the consortium. Finally, *i*BCc98 predicts that *B. aphidicola* BCc is only able to synthesize some membrane lipids, as has been previously described (Lamelas et al., 2011a).

As in *S. symbiotica* SCc, the genome of *B. aphidicola* BCc has maintained the genes for glycolysis and acetate fermentation, but with a higher energetic efficiency, producing 5 ATP mols per glucose mol. This is due to the fact that *B. aphidicola* BCc lacks genes for PTS transporters and hexokinases, and therefore the model assumes that the sugar molecules must be pre-phosphorylated when imported, and no ATP is used for this step. However, *B. aphidicola* BCc has lost the ATP synthase genes, and is only able to synthesize ATP at substrate level. Interestingly, this loss is not coupled to that of the respiratory chain complexes NADH-dehydrogenase and cytochrome *bo*$_3$, and the explanation for such a disparity is not clear. According to the *i*BCc98 model, these systems may be used to dissipate reducing power produced during glycolysis and other processes. However, this generates a balance problem, where the generation of 6 protons per glucose molecule during glycolysis (1) and the posterior hydrolysis of the ATP molecules generated in that pathway (5), is not enough to compensate the loss of cytosolic protons caused by the respiration of oxygen (4) and the proton pumping (8) carried out during the oxidation of the 4 NADH molecules generated per glucose molecule during glycolysis.

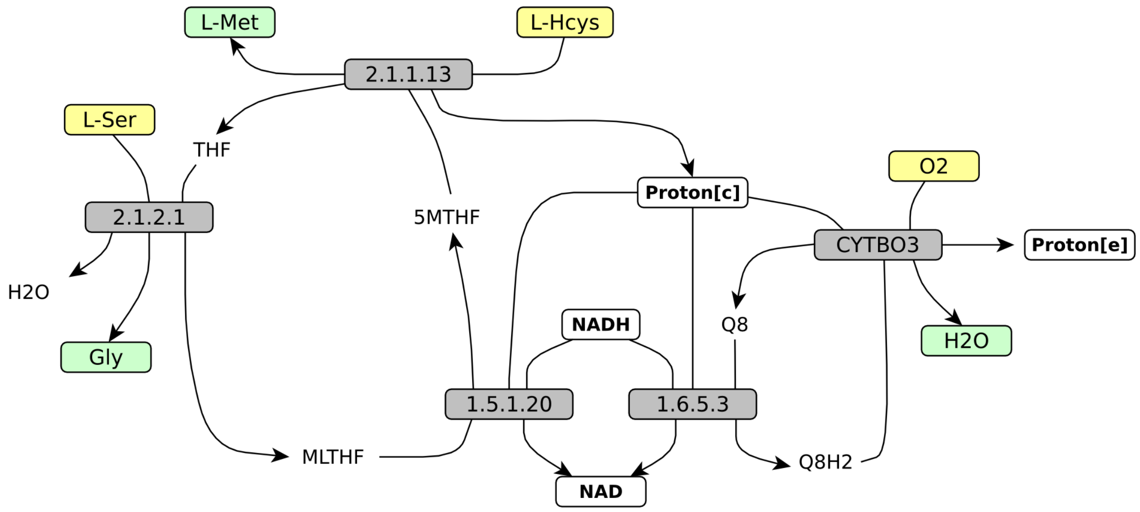

Figure 1. Metabolic diagram showing the predicted reactions involved in the regeneration of NADH and proton balance in i**BCc98.** *The diagram includes the enzymatic activities of the folate cycle, the NADH dehydrogenase complex and the cytochrome oxidase. Grey, yellow and green rectangles represent reactions, substrates and products, respectively. [c] and [e] denote cytosolic and extracellular protons, respectively. All other metabolites are cytosolic.*

The analysis of optimal fluxes that maximize ATP synthesis using *i*BCc98 predicts that only 1 out of the 4 NADH molecules is oxidized by the respiratory chain, while the other 3 are oxidized in the folate cycle, with consumption of 3 protons. This pathway (figure 1) balances both the NADH molecules and the protons, but consumes homocysteine and serine, and produces glycine and

methionine, which need to be excreted. This pattern is avoided with the inclusion in the model of a free proton reentry flux, indicating that the overproduction of methionine and glycine is unambiguously coupled to the proton gap. On the other hand, if the import of homocysteine and serine is artificially bounded to zero, and FBA is used to obtain the flux distribution optimizing ATP production, an efficiency of only about 1 ATP mol is produced per glucose 6-phosphate mol. Notably, this optimal flux distribution over-produces valine, 8AONN and anthranilate, effect which is again avoided if protons are allowed to traverse the membrane freely. These results suggest an unorthodox role for the respiratory chain in metabolic coupling with the overproduction of metabolites that are essential for the host or *S. symbiotica* SCc.

The effect caused by proton balance in GEMs is generally low due to the size of these networks. However, in endosymbionts and other small networks, it may generate notorious consequences. In *i*BCc98, for instance, it considerably reduces the versatility of the metabolism of *B. aphidicola* BCc by coupling presumably independent processes, such as ATP synthesis and the folate cycle, and over-producing amino acids. Although this metabolic organization would be clearly disadvantageous for a free-living organism, for an endosymbiont member of a nutritional symbiosis it may be selected for at the level of the host. Indeed, a similar behaviour has been described recently as applied to the whitefly endosymbiont *P. aleyrodidarum* (Calle-Espinosa et al., 2016), where the growth of the organism is coupled with the overproduction of amino acids and carotenoids as a consequence of its low energetic capabilities. This phenomenon might play a relevant role in the evolution of nutritional endosymbiosis but it may also represent a methodological artefact as a consequence of the lack of knowledge on how to formulate in such a model the transport of protons through the membrane. One possibility would be the use of protons for the transport of compounds against their gradients. Although the scarcity of annotated transporters in the *B. aphidicola* BCc genome (Charles et al., 2011) does not seem to support this scenario, this problem falls within a more general umbrella, whereby the nature of the cell envelope (including both the membrane composition and the transport systems) of endosymbionts is largely unknown and might rely on contributions from the host (McCutcheon, 2016).

Construction of a biomass equation for the aphid *C. cedri*

The stoichiometric coefficients for the set of amino acids needed for the host growth were estimated from the available data on protein composition available in literature. Two datasets are available, for *Aphis fabae* (Douglas et al.,2001) and *Acyrtosiphon pisum* (Russell et al., 2014), both of which are reported in Table 3. As shown in Fig. 2, the amino acid compositions of the two aphids correlate strongly, with a correlation coefficient $r = 0.85$ ($p = 7.8 \times 10^{-6}$). As stated in the Main Text, we use for *C. cedri* the more recent values from *A. pisum* (Russel et al.,2014).

Table 3. Two dataset of the proteome composition (in molar percentage composition). *The second and third columns show the values of the measured proteome composition for Aphis fabae (Douglas et al., (2001) and Acyrthosiphon pisum (Russell et al., 2014) respectively. Asx and Glx values correspond to the combined values of Gln+Glu and Asn+Asp, respectively, as in reported in the original publication (Douglas et al. (2001). The values for these 4 amino acids were measured independently in Russell et al. (2014) but herein they were merged for consistency in the comparison.*

**Amino acid**	**%mol (*A. fabae*)**	**%mol (*A. pisum*)**
Alanine	5.6	7.8
Arginine	2.6	4.5
Asx	12.9	10.5
Cysteine	1.7	1.2
Glx	13.3	11.3
Glycine	4.2	6.7
Histidine	0.9	1.8
Isoleucine	6.5	5.9
Leucine	10.5	8.4
Lysine	8.6	7.7
Methionine	2.4	2.3
Phenylalanine	4.6	3.8
Proline	6.3	4.6
Serine	4.6	6.9
Threonine	6.1	3.2
Tryptophan	0.5	5.4
Tyrosine	1.1	0.8
Valine	7.6	7.2

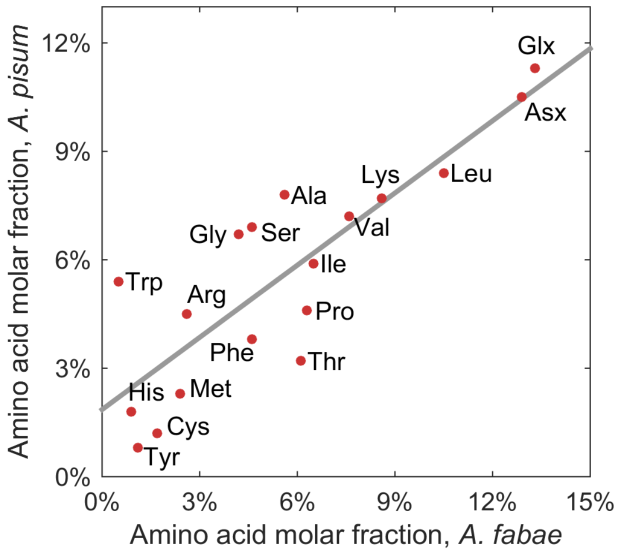

Figure 2. Scatter plot of the datasets corresponding to the composition of the *A. fabae* (Douglas et al., 2001) and *A. pisum* (Russell et al., 2014) proteomes. The solid line correspond to the regression line, with slope = 0.666 and intercept = 1.86. The correlation coefficient between the two sets is $r = 0.85$ (p = 7.8 x 10^{-6}).

Definition of a meta-biomass equation to simulate insect-consortium growth

Different strategies have been developed for the analysis of microbial communities, allowing the simulation of different kinds of interactions (Stolyar et al., 2007; Taffs et al., 2009; Vallino, 2003). However, endosymbiotic consortia are peculiar in this regard, given the mutual interdependency between the members of the consortium, due to the metabolic complementation established through long co-evolutionary processes. On one hand, correlations of aphid dry weight and marker gene abundance have shown that the growth dynamics of the aphid *Schizaphis graminum* is strongly linked to those of their primary symbiont, *B. aphidicola* Sg, especially during the first developmental stages (Baumann and Baumann, 1994; Mira and Moran, 2002). Table 1 shows the results on the experimental measurements on the growth kinetics of the aphid *S. graminum* and its endosymbiont *B. aphidicola* BSg.

The experimental results depicted in Table 4 show a close relationship between the growth of the aphid mass and the increase in the number of copies of the endosymbiont 16S rRNA gene. Thus, if it is assumed that the number of copies of the 16S rRNA gene allows to estimate the number of symbionts, this measure can be compared to the mass of the aphid during the growth phase. In fact, the Pearson correlation test indicates that the correlation between the two variables is ~ 0.97 with a p-value of 10^{-7}. Although the number of copies of the 16S rRNA gene may present problems for the estimation of abundances in microbial communities, since the number of copies of the gene can vary between genomes of different species (Větrovský and Baldrian, 2013), the considered data only include the abundance of a single bacterial species, whose genome only encodes for a single copy of the 16S rRNA gene. Moreover, studies of the estimation of the number of endosymbionts performed by manual counting of individuals in different sections of insect samples, observed by electron

microscopy, have allowed to estimate the population of bacteria directly (Mira and Moran, 2002) . The population sizes measured by Mira and Moran (2002) are in agreement with values previously estimated by molecular methods (Baumann and Baumann, 1994), as well as with other similar studies (Humphreys and Douglas, 1997). This indicates that the number of copies of the 16S rRNA gene can be used as an estimator of the number of individuals. More recently, flow cytometry, combined with histological and imaging techniques, have been used to show exponential growth of the symbiont during the development of the host (Simonet et al., 2016). On the other hand, experimental analysis of two synthetic *E. coli* strains, auxotrophic for different amino acids and mutually interdependent, showed that at pseudo-stationary state both strains grow at similar rates (Kerner et al., 2012).

Table 4. Growth characteristics of the aphid S. graminum and its endosymbiont B. aphidicola BSg. *Columns includes the following data: No. Days; No. aphid count; No. samples; aphid average weight (µg); protein and DNA concentration (µg/aphid); No. of Buchnera 16S rRNA copies / pg de DNA; No. Buchnera16S rRNA copies (10^6) / aphid. Data reproduced from Baumann and Baumann (1994). Highlighted columns in gray depict the values used to compute Pearson correlation coefficient.*

Days	Aphids	Samples	Aphid wt. (ug)	[Protein]	[DNA]	rrs / pg aphid DNA	N rrs (10e6) /aphid
1	1530	8	24 ± 2.9	1.8	0.054	3.74 ± 0.12	0.2
2	322	3	44.5 ± 1		0.099	3.79 ± 0.42	0.38
3	863	8	68.6 ± 1.9	5.2	0.148	3.73 ± 0.21	0.55
4	191	3	111.3 ± 6.7	7.8	0.246	2.28 ± 0.21	0.56
5	271	5	178 ± 5.3	12.8	0.452	2.11 ± 0.27	0.95
6	157	3	242.7 ± 8.7		0.788	2.62 ± 0.15	2.07
7	232	5	324.2 ± 15	25.8	1.088	3.5 ± 0.25	3.81
8	155	3	437 ± 5.6	35.4	1.433	3.67 ± 0.2	5.26
9	143	3	497.3 ± 12.7	41.7	1.667	3.36 ± 0.21	5.6
10	137	3	535 ± 7.2	45.4			
11	188	3	541 ± 12.1	46.8	1.64	3.06 ± 0.17	5.02
12	162	4	541.3 ± 10.8	47.1	1.681	3.06 ± 0.08	5

Altogether, it can be assumed that the growth rates of the members of a mutualistic consortium are coupled. An objective function that allows the calculation of metabolic capabilities of the consortium during exponential growth of the symbionts would resemble that of the biomass equation of a free-living bacterium. In such cases, a meta-biomass equation can be used to describe the growth of the system (Stolyar et al., 2007; Calle-Espinosa et al., 2016), which would especially apply during the development of the host. In this equation, the biomass equations of the symbionts would be combined with a biomass equation for the host. Therefore, the meta-biomass equation is a fictitious reaction consuming auxiliary metabolites representing the biomass of each member of the consortium in fixed stoichiometric proportions (see main text). Since the exact contribution of the symbionts to the cedar aphid biomass is unknown, we used data obtained from *S. graminum* and *A. pisum* that showed that their symbionts represent 5-15% of the system's biomass (Baumann et al., 2006; Whitehead and Douglas, 1993). Thus, we modeled the proportion between biomass of the symbionts and the host to be 1:9.

Bibliography

Baumann, L., Baumann, P., 1994. Growth Kinetics of the Endosymbiont Buchnera aphidicola in the Aphid Schizaphis graminum. Appl. Environ. Microbiol. 60, 3440–3443.

Baumann, P., Moran, N.A., Baumann, L., 2006. The Prokaryotes. Springer New York, New York, NY. doi:10.1007/0-387-30741-9

Calle-Espinosa, J., Ponce-de-Leon, M., Santos-Garcia, D., Silva, F.J., Montero, F., Peretó, J., 2016. Nature lessons: The whitefly bacterial endosymbiont is a minimal amino acid factory with unusual energetics. J. Theor. Biol. 407, 303–317.

Charles, H., Balmand, S., Lamelas, A., Cottret, L., Pérez-Brocal, V., Burdin, B., Latorre, A., Febvay, G., Colella, S., Calevro, F., Rahbé, Y., 2011. A genomic reappraisal of symbiotic function in the aphid/Buchnera symbiosis: reduced transporter sets and variable membrane organisations. PLoS One 6, e29096. doi:10.1371/journal.pone.0029096.

Douglas, A. E., Minto, L. B., Wilkinson, T. L., 2001. Quantifying nutrient production by the microbial symbionts in an aphid. J. Exp. Biol. 204, 349-358.

Gosalbes, M.J., Lamelas, A., Moya, A., Latorre, A., 2008. The striking case of tryptophan provision in the cedar aphid Cinara cedri. J. Bacteriol. 190, 6026–6029. doi:10.1128/JB.00525-08

Humphreys, N.J., Douglas, A.E., 1997. Partitioning of symbiotic bacteria between generations of an insect: A quantitative study of a Buchnera sp. in the pea aphid (Acyrthosiphon pisum) reared at different temperatures. Appl. Environ. Microbiol. 63, 3294–3296.

Kerner, A., Park, J., Williams, A., Lin, X.N., 2012. A programmable Escherichia coli consortium via tunable symbiosis. PLoS One 7, e34032. doi:10.1371/journal.pone.0034032

Mira, A., Moran, N.A., 2002. Estimating population size and transmission bottlenecks in maternally transmitted endosymbiotic bacteria. Microb. Ecol. 44, 137–43. doi:10.1007/s00248-002-0012-9

Russell, C. W., Poliakov, A., Haribal, M., Jander, G., van Wijk, K. J., Douglas, A. E., 2014. Matching the supply of bacterial nutrients to the nutritional demand of the animal host. Proc. Royal Soc. London B: Biol. Sci. 281, 20141163. doi:10.1098/rspb.2014.1163

Simonet, P., Duport, G., Gaget, K., Weiss-Gayet, M., Colella, S., Febvay, G., Charles, H., Viñuelas, J., Heddi, A., Calevro, F., 2016. Direct flow cytometry measurements reveal a fine-tuning of symbiotic cell dynamics according to the host developmental needs in aphid symbiosis. Sci. Rep. 6, 19967. doi:10.1038/srep19967

Stolyar, S., Van Dien, S., Hillesland, K.L., Pinel, N., Lie, T.J., Leigh, J. a, Stahl, D. a, 2007. Metabolic modeling of a mutualistic microbial community. Mol. Syst. Biol. 3, 92. doi:10.1038/msb4100131.

Taffs, R., Aston, J.E., Brileya, K., Jay, Z., Klatt, C.G., McGlynn, S., Mallette, N., Montross, S., Gerlach, R., Inskeep, W.P., Ward, D.M., Carlson, R.P., 2009. In silico approaches to study mass and energy flows in microbial consortia: a syntrophic case study. BMC Syst. Biol. 3, 114. doi:10.1186/1752-0509-3-114

Vallino, J.J., 2003. Modeling microbial consortiums as distributed metabolic networks. Biol. Bull. 204, 174–9.

Větrovský, T., and Baldrian, P. (2013). The Variability of the 16S rRNA Gene in Bacterial Genomes and Its Consequences for Bacterial Community Analyses. PLoS One 8, e57923. doi:10.1371/journal.pone.0057923.

Whitehead, L.F., Douglas, A.E., 1993. Populations of Symbiotic Bacteria in the Parthenogenetic Pea Aphid (Acyrthosiphon pisum) Symbiosis. Proc. R. Soc. B Biol. Sci. 254, 29–32. doi:10.1098/rspb.1993.0122.
