# Supplementary material for: Determinism and Contingency Shape Metabolic Complementation in an Endosymbiotic Consortium
Source: Front Microbiol. 2017 Nov 22;8:2290. doi: 10.3389/fmicb.2017.02290 (PMC5702781; doi:10.3389/fmicb.2017.02290)
Supplement: Supplementary Text S2 — Extended Materials and Methods. The text includes details on the annotated genomes, constraint-based methods employed, as well as the reconstruction of the plausible ancestor model of C. cedri used as a reference point in the reduction experiments. [file TextS2.PDF]

Supplementary Text S2 - Extended Material and Methods

A genome-scale study of metabolic complementation in endosymbiotic consortia: the case of the cedar aphid.

Miguel Ponce-de-Leon[1*], Daniel Tamarit[2], Jorge Calle-Espinosa[1], Matteo Mori[3], Amparo Latorre[4,5], Francisco Montero[1], Juli Pereto[5,6*]

* Corresponding authors:
- Miguel Ponce-de-Leon: migponce@ucm.es
- Juli Pereto: pereto@uv.es

Genome annotations

Table S2.1 described the genome annotation of the primary and co-primary endosymbiont of the cedar aphid, named *B. aphidicola* BCc and *S. symbiotica* SCc.

Table S2.1 Description of the annotation of **S. symbiotica SCc.** ***and*** **B. aphidicola BCc genomes.**

Organism	Replicon	Genes	CDSs	RNAs	Pseudogenes	Length (bps)
S. symbiotica SCc	NC_016632 (chr)	712	672	40	59	1,762,765
	**Total:**	**712**	**672**	**40**	**59**	**1,762,765**
B. aphidicola BCc	NC_008513 (chr)	430	358	72	3	416,380
	NC_011878 (pl)	5	5	0	0	6,054
	*EU660486.1 (pl)	2	2	0	0	2,795
	**Total:**	**438**	**365**	**72**	**3**	**425,229**

* The plasmid EU660486.1 was added manually to a local version of the *B. aphidicola* BCc PGDB
Abbreviations: chr. (chromosome); pl (plasmid).

Constraint-based modeling methods

Most of the constraint-based methods used in the present work correspond to the current implementation found in COBRApy, the python-based implementation of the OpenCobra toolbox (Ebrahim et al., 2013). The biosynthetic capabilities of the endosymbionts were evaluated through the calculation of the minimal requirements needed for the individual synthesis of each of the biomass components. Moreover, the energetic capabilities are evaluated through the analysis of flux distribution that optimizes ATP production computed using FBA.

Flux Balance Analysis

The Flux Balance Analysis (FBA) approach is a computational approach which allows the calculation of optimal flux distribution on GEMs. It is formulated as the following standard linear program:

$$
\begin{aligned}
&Max\ Z = v_{Biomass}\\
&s.t.\\
&N \cdot \vec{v} = 0\\
&\beta_j \leq v_j \leq \alpha_j \forall j \epsilon J\\
&\alpha_j = 0 \ \ \forall j \in J_{Irr}
\end{aligned}
\qquad \textbf{(1)}
$$

where $\boldsymbol{N}$ correspond to the *m x n* stoichiometric matrix; $\boldsymbol{v}$ is a flux distribution, *i.e.* a *n x 1* vector where each entries $\boldsymbol{v_j}$ corresponds to the flux value of the reaction $\boldsymbol{j}$; $\boldsymbol{\beta_j}$ and $\boldsymbol{\alpha_j}$ are the lower and upper bounds of the reaction, respectively; $\boldsymbol{J}$ is the set of reaction indexes and $\boldsymbol{J_{Irr}} \subset \boldsymbol{J}$ is the subset of irreversible reaction indexes.

Minimization of the Metabolic Adjustment

The Minimization of the Metabolic Adjustment (MOMA) is a computational method to calculate the flux distribution on a GEM after a genetic perturbation is introduced, *e.g.* a gene knockout (Segre et al. 2002). MOMA is formulated as the following quadratic program:

$$
\begin{aligned}
&Min\ Z = \frac{1}{2} v^T Q v \ - \ \boldsymbol{w}^{\boldsymbol{T}} \cdot v\\
&s.t.\\
&N \cdot \vec{v} = 0\\
&\beta_j \leq v_j \leq \alpha_j \forall j \epsilon J\\
&\alpha_j = 0 \ \ \forall j \in J_{Irr}
\end{aligned}
\qquad \textbf{(2)}
$$

Computation of Enzyme Subsets

An *Enzyme Subset* (ES) (Pfeiffer et al. 1999) or *Full Coupling Set* (Burgard et al., 2004) is a group of enzymes that operate together in fixed flux proportions in any flux distribution under steady state. Computation of ES, has been described elsewhere (see Pfeiffer et al., 1999), and requires the calculation of a basis of the null space, or *kernel*, of the stoichiometric matrix $\boldsymbol{N}$. If the vector of the kernel is arranged as columns of a matrix $\boldsymbol{K}$, then, each ES correspond to a subset of linear dependent rows of $\boldsymbol{K}$. Since in a GEM the biomass reactions imposes a stoichiometric constraint that link together the output fluxes of many compounds, a common pre-processing step consists in relaxing this constraint by allowing each biomass component to be drained from the system in an independent way (Burgard et al., 2004).

Reconstruction of a plausible ancestral consortium model

The reconstruction of the hypothetical ancestral consortium was conducted by adding to each compartment (*i.e.* endosymbiont) the missing enzymatic activities of the biosynthetic pathways of tryptophan, THF and biotin. According to BioCyc 19.0, *S. symbiotica* SCc is the only *Serratia* strain (out of 17 strains in 9 different species) lacking the genes for the synthesis of shikimate from erythrose-4-phosphate (E4P). Therefore, it is reasonable to assume they were present in an ancestor of this lineage, and we added the activities encoded by the genes *aroH*, *aroB*, *aroQ* and *aroE* to the *S. symbiotica* SCc compartment. Finally, the transport fluxes limits for shikimate, chorismate, anthranilate and tryptophan were modified, so these metabolites could be freely exchanged between both symbionts. Furthermore, the synthesis of tryptophan from chorismate requires 6 reactions, and the latter is produced from E4P in 7 steps. The genes that encode the enzymes that catalyze these

reactions are distributed between the two symbionts: *B. aphidicola* BCc contains the genes necessary for the synthesis of anthranilate from E4P, while *S. symbiotica* SCc has the ones that encode the activities for the synthesis of chorismate from shikimate, and of tryptophan from anthranilate (see Table 1 in the main text). Thus, in order to reconstruct the ancestral consortium model, we added the activities encoded in the genes *trpABCD* to the *B. aphidicola* BCc compartment, and the ones encoded by the genes *trpEG* to the *S. symbiotica* SCc compartment.

Genes that belong to the same FCS can be treated as equivalent since the removal of any of them will inactivate the entire FCS. Thus, it is only necessary to evaluate one candidate gene per FCS, which considerably reduces the number scenarios to evaluate. Thus, the grouping was done by mapping the genes into the different ESs, through the GRP association rules. As shown in table S2.2, the three biosynthetic pathways analyzed involving a total of 32 genes can be grouped in 7 modules, which in the case of *i*BSCc$_{\text{Ances}}$ each module is present in both endosymbionts. Since, at least one of the two copies for each ES needs to be functional, the maximum number of ES losses is 7. That is to say, the minimal designs that allow the biosynthesis of the three compounds are the combinations of seven ES that do not include redundant ES.

Bibliography

Burgard, A. P., Nikolaev, E. V, Schilling, C. H., and Maranas, C. D. (2004). Flux coupling analysis of genome-scale metabolic network reconstructions. Genome Res. 14, 301–12. doi:10.1101/gr.1926504.

Ebrahim, Ali, Joshua A. Lerman, Bernhard O. Palsson, Daniel R. Hyduke, and Hyduke DR Ebrahim A, Lerman JA, Palsson BO. 2013. “COBRApy: COnstraints-Based Reconstruction and Analysis for Python.” BMC systems biology 7(1):74.

Pfeiffer, T., Sánchez-Valdenebro, I., Nuño, J. C., Montero, F., and Schuster, S. (1999). METATOOL: for studying metabolic networks. Bioinformatics 15, 251–257.

Segrè, Daniel, Dennis Vitkup, and George M. Church. 2002. “Analysis of Optimality in Natural and Perturbed Metabolic Networks.” Proceedings of the National Academy of Sciences of the United States of America 99(23):15112–17.
