# Supplementary figures and images for: Determinism and Contingency Shape Metabolic Complementation in an Endosymbiotic Consortium
Source: Front Microbiol. 2017 Nov 22;8:2290. doi: 10.3389/fmicb.2017.02290 (PMC5702781; doi:10.3389/fmicb.2017.02290)

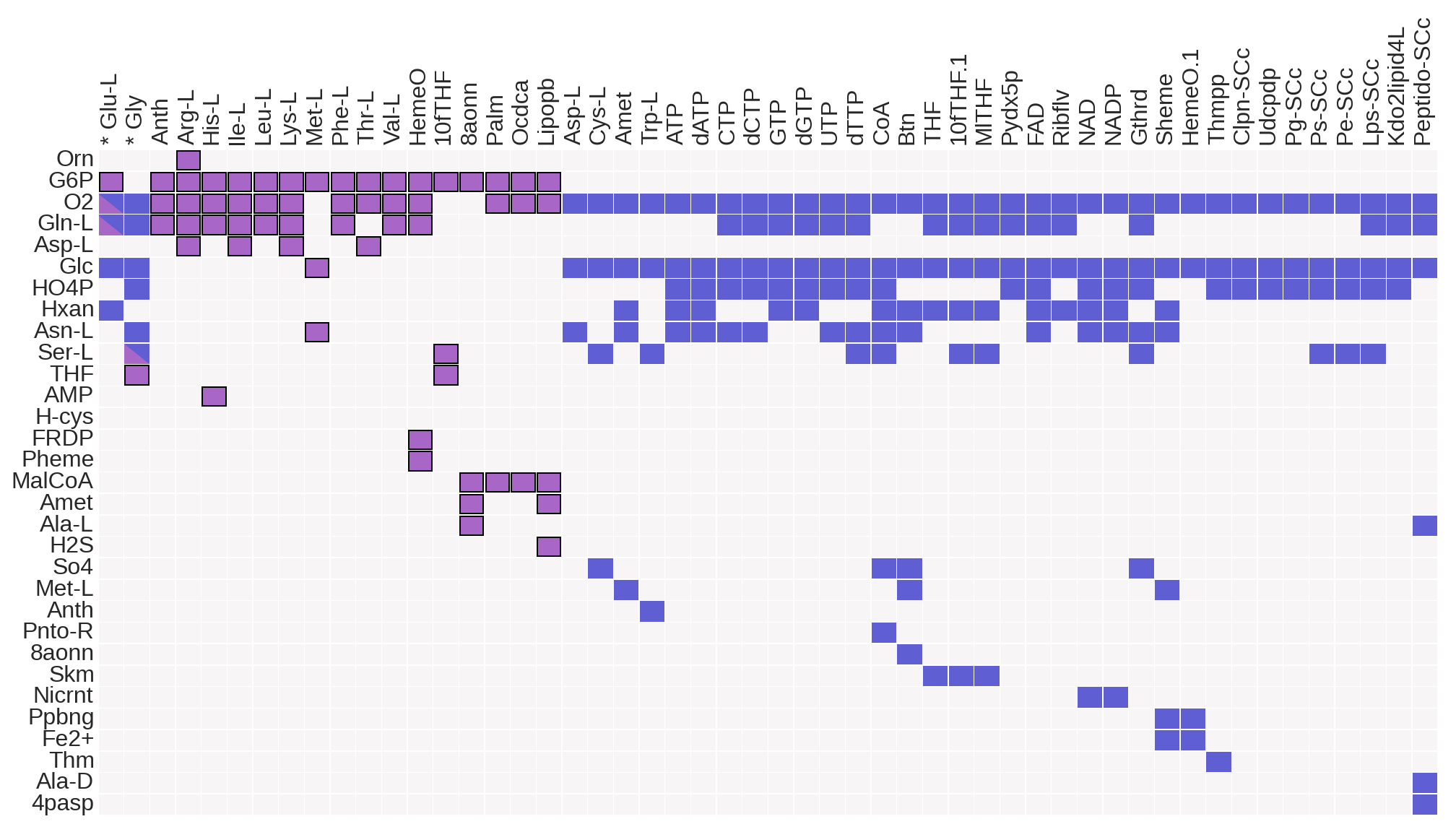

Supplement: Supplementary Figure S1 — Conversion map predicted by iBCc98 and iSCc236. Rows represent imported compounds, and columns, biomass components synthesized by each organism. Colored squares in the same columns indicate the set of compounds required for the biosynthesis of a component. Purple and blue squares correspond to capabilities predicted by iBCc98 and iSCc236, respectively. Marked columns (*) correspond to compounds produced by both bacteria. [file Image1.JPEG]

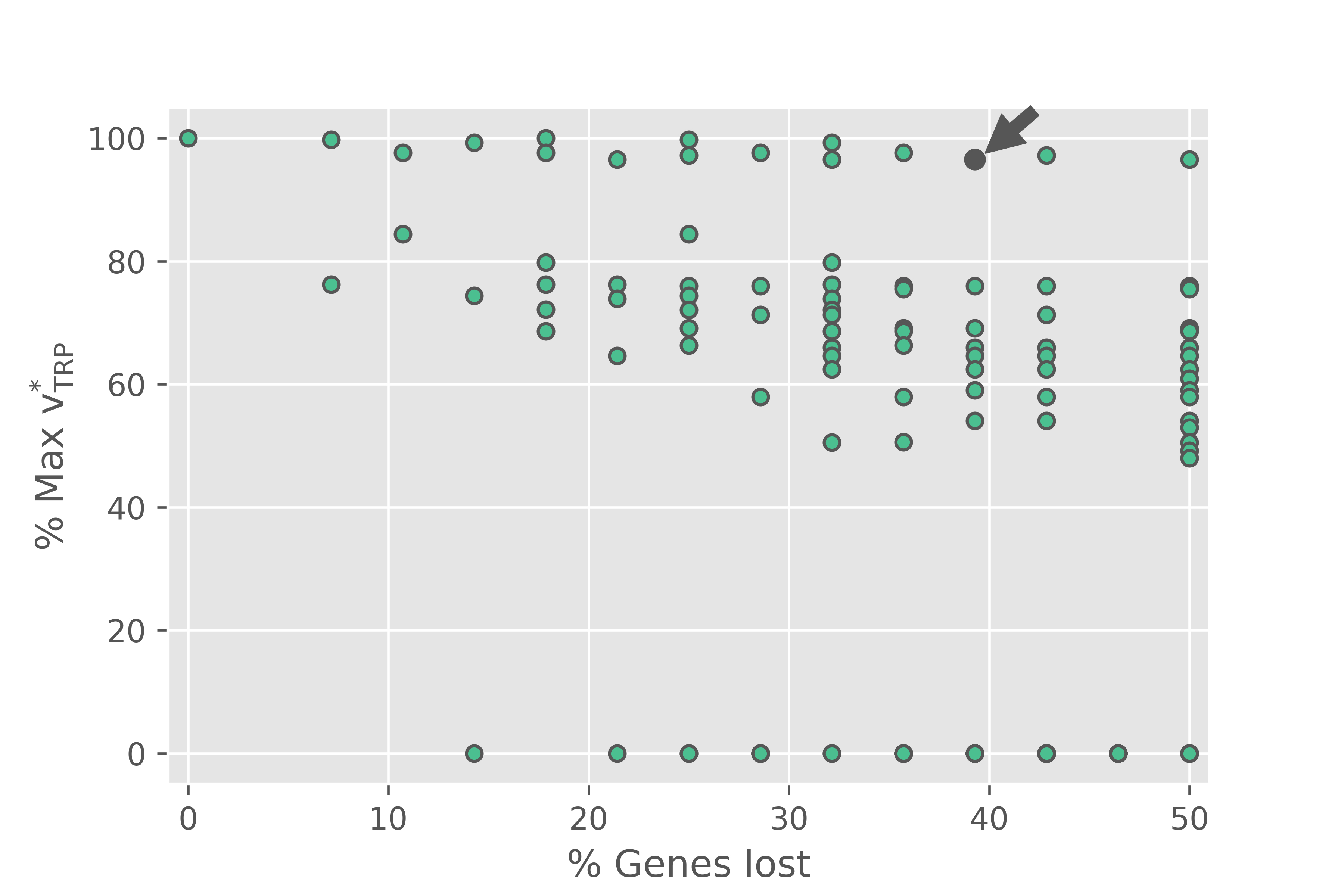

Supplement: Supplementary Figure S2 — Optimal production rates of tryptophan for the reduced gene loss and retention experiments. Normalized optimal production rate of tryptophan with respect to the optimal value exhibited. The small arrow denotes the case of iBSCc, i.e., the cedar aphid consortium. [file Image2.JPEG]

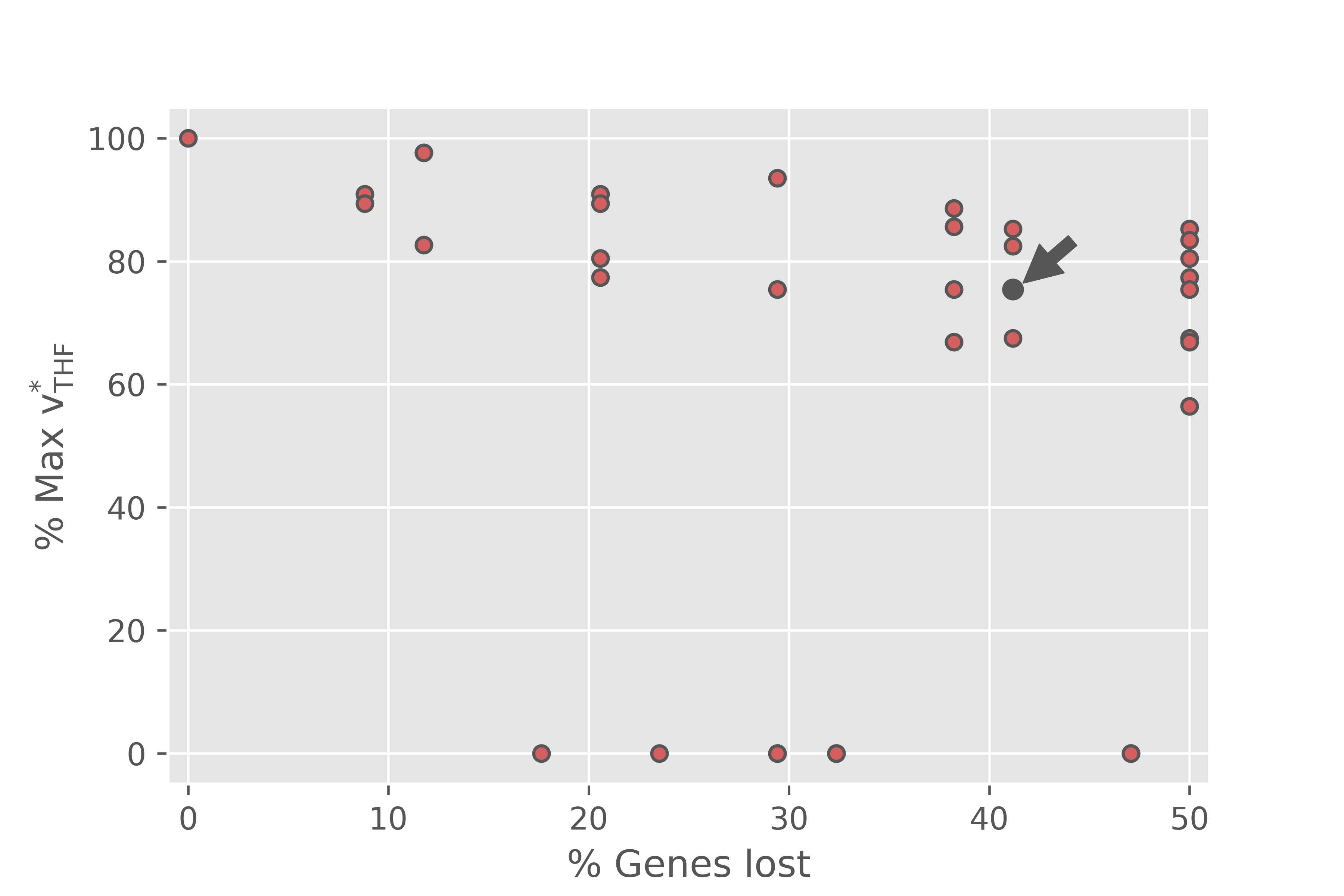

Supplement: Supplementary Figure S3 — Optimal production rates of tetrahydrofolate for the reduced gene loss and retention experiments. Normalized optimal production rate of tetrahydrofolate with respect to the optimal value exhibited. The small arrow denote the case of iBSCc, i.e., the cedar aphid consortium. [file Image3.JPEG]

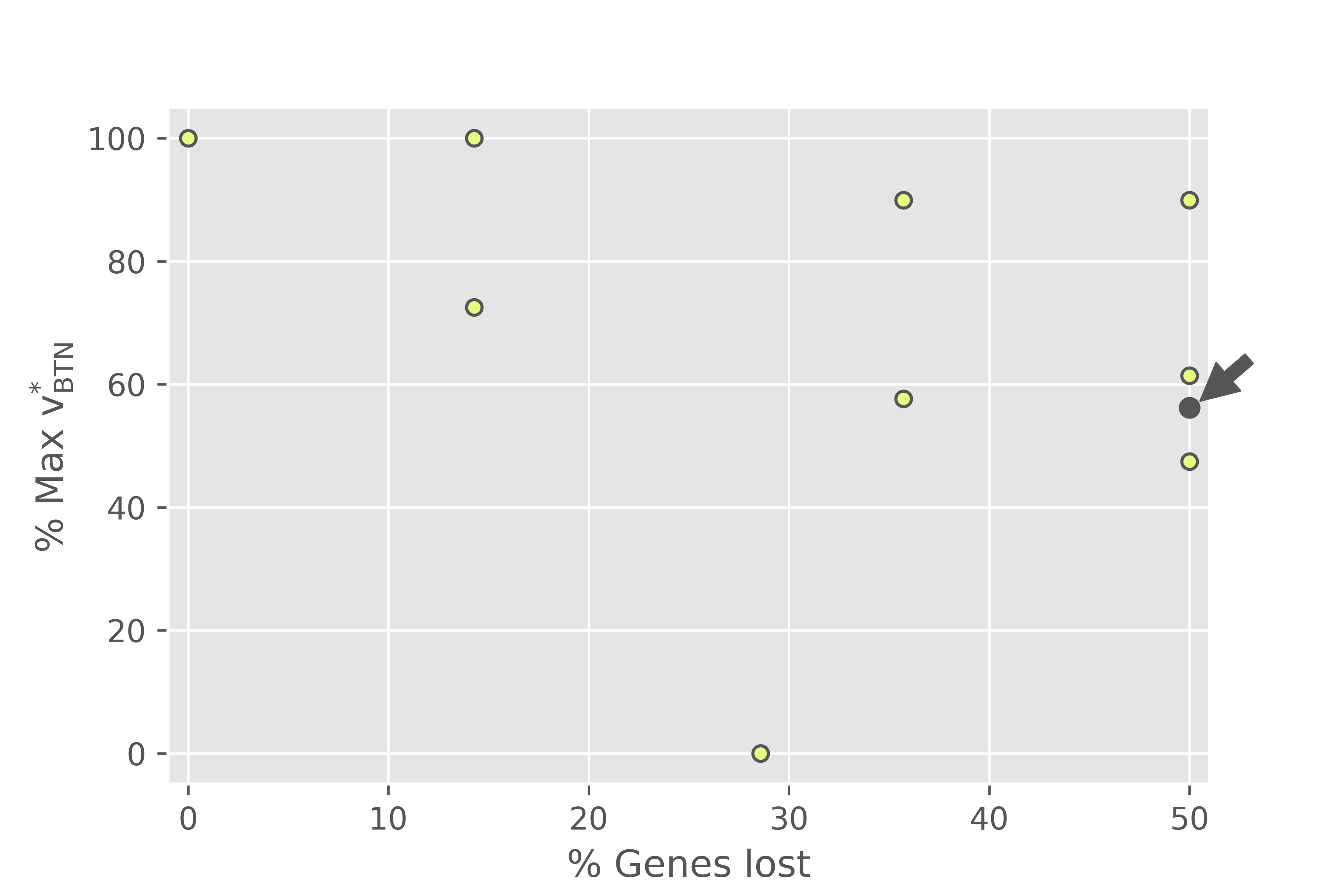

Supplement: Supplementary Figure S4 — Optimal production rates of biotin for the reduced gene loss and retention experiments. Normalized optimal production rate of biotin with respect to the optimal value exhibited. The small arrow denote the case of iBSCc, i.e., the cedar aphid consortium. [file Image4.JPEG]
